# PD81 Applying The Innovation Of Health Technology Assessment Methods Framework To Develop The European Digital Health Technology Assessment (EDiHTA) Framework

**DOI:** 10.1017/S0266462325102080

**Published:** 2025-12-29

**Authors:** Emmanouil Tsiasiotis, Rossella Di Bidino, Fruzsina Mezei, Michele Basile, Livio Battaglia, Valentina Strammiello, Wija Oortwijn, Dario Sacchini, Americo Cicchetti

## Abstract

**Introduction:**

The EDiHTA project aims to deliver an innovative health technology assessment (HTA) framework for digital health technologies (DHT) that integrates existing methods with new ones to inform decision-making across Europe at different levels. In the development phase of the EDiHTA framework, it has been essential to identify all relevant stakeholders and their roles in the innovation process, while also capturing their needs and requirements.

**Methods:**

The Innovation of Health Technology Assessment Methods framework was applied to identify relevant stakeholders and their needs and requirements regarding the design of the EDiHTA framework. Once stakeholders and their roles (practitioners or beneficiaries) were identified, their needs were assessed by reviewing existing and new HTA methodologies for DHT as used in 17 countries across Europe. Future scenarios were examined and gaps to be addressed were determined. Various methods were used to elicit the views of different stakeholders (policymakers, HTA agencies and bodies, industry, healthcare professionals, patients). including literature reviews, two online surveys, semi-structured online interviews, and focus groups.

**Results:**

We engaged policymakers from 15 European countries; 15 HTA agencies from nine European countries; 29 developers (startups and big companies) from 10 different European markets; 14 healthcare professionals from nine European countries plus eight from Brazil, Canada, and the USA; and 15 patient representatives from 10 European countries. The analysis highlighted the heterogeneity among European health systems in assessing, implementing, and reimbursing DHT. All stakeholder groups emphasized the need for a harmonized HTA framework to assess DHT efficiently and accurately, including elements specific to DHT, such as cybersecurity, economic and organizational impact, and patient-centered outcomes.

**Conclusions:**

There is a clear need among European stakeholders for an agile, flexible, and multidimensional HTA framework that facilitates stepwise assessments across the technology life cycle and involves all relevant stakeholders in the assessment to inform decision-making. The stakeholder interaction also identified new HTA topics and domains essential for effectively addressing the unique challenges of DHT within the EDiHTA framework.

